# *Phormia regina* Fly as Vector for *Ignatzschineria* spp. Bacteremia in Persons Experiencing Homelessness, Canada, 2025

**DOI:** 10.3201/eid3207.251914

**Published:** 2026-07

**Authors:** Emma C.L. Finlayson-Trick, Awatif F. Alenazi, Yi Zhen Jia, Michael Payne, Gordon Ritchie, Aleksandra Stefanovic, Christopher F. Lowe, Samuel D. Chorlton, Nancy Matic, Patrick Tang, Victor Leung, David Harris, Marc G. Romney

**Affiliations:** University of British Columbia, Vancouver, British Columbia, Canada (E.C.L. Finlayson-Trick, A.F. Alenazi, Y.Z. Jia, M. Payne, G. Ritchie, A. Stefanovic, C.F. Lowe, N. Matic, P. Tang, V. Leung, D. Harris, M.G. Romney); St. Paul’s Hospital, Vancouver (M. Payne, G. Ritchie, A. Stefanovic, C.F. Lowe, N. Matic, P. Tang, V. Leung, D. Harris, M.G. Romney); BugSeq Bioinformatics, Inc., Vancouver (S.D. Chorlton)

**Keywords:** Phormia regina, Ignatzschineria, bacteria, bacterial infections, bacteremia, vector-borne infections, zoonoses, whole-genome sequencing, myiasis, One Health, homelessness, Vancouver, Canada

## Abstract

*Ignatzschineria* spp. bacteria are emerging pathogens whose vectors historically have not been clearly identified. We used molecular methods to establish a relationship between the black blow fly (*Phormia regina*) and human *Ignatzschineria* bacteremia in persons experiencing homelessness in Vancouver, British Columbia, Canada, validating a novel transmission pathway in a vulnerable urban population.

*Ignatzschineria* bacteria are increasingly recognized as a cause of human infection (*1*). Four species have been described to date: *I. indica*, *I. larvae*, *I. ureiclastica*, and *I. cameli* (*1*). Historically, species associated with human wound and blood infection have shown a geographic distribution; *I. indica* predominates in North America, and *I. larvae*/*I. ureiclastica* predominates in Europe (*1*). The gram-negative, aerobic, nonmotile, and oxidase- and catalase-positive bacteria were originally isolated from the larvae and adult gastrointestinal tracts of *Wohlfahrtia magnifica* parasitic flies (*2*). Experimental and ecologic studies suggest that *Ignatzschineria* are part of the larvae microbiome through a combination of vertical transmission and environmental acquisition during feeding (*3*–*5*). Ultimately, host identity appears to shape overall abundance; blow fly species such as *Phormia regina* have a high abundance of *Ignatzschineria* (Appendix Figure 1) (*4*).

Although *W. magnifica* flies primarily cause myiasis in animals, blow flies are associated with facultative myiasis in humans (*6*). At least 3 case reports of *Ignatzschineria* bacteremia in humans have implicated the green blow fly (*Lucilia sericata*), but the methods used for identification have primarily relied on epidemiology and morphologic features (*1*,*7*). In general, the specific fly vectors associated with human urban myiasis are seldom confirmed molecularly.

We describe 2 cases of *Ignatzschineria* bacteremia in persons experiencing homelessness (PEH) in Vancouver, British Columbia, Canada. We used genomic approaches to characterize the bacterial isolates from both cases and performed vector identification on the fly associated with 1 of the cases. We obtained written consent from both patients for publication of their cases.

## The Study

**Figure 1:**
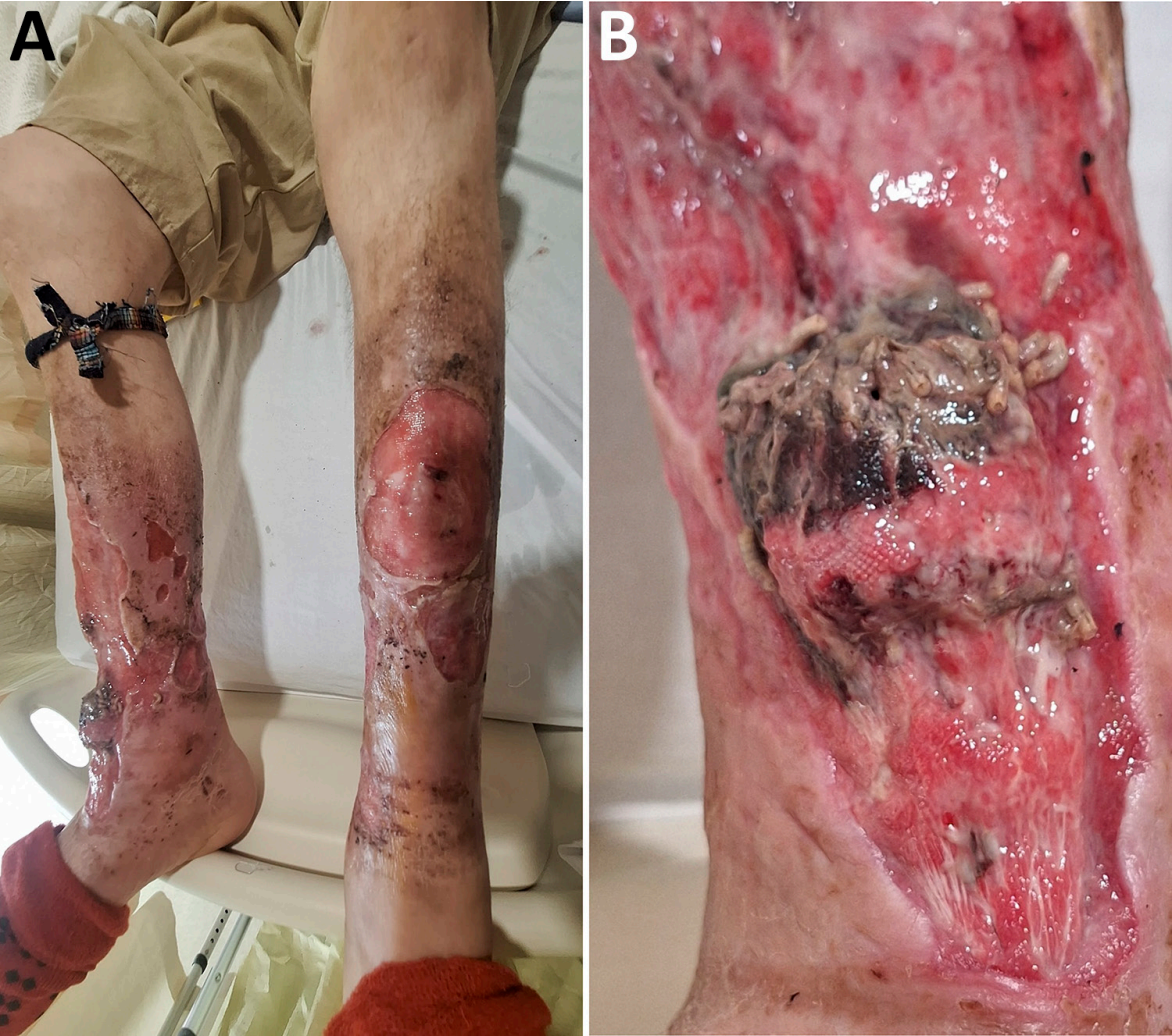
Leg wounds in case-patient 1 in pair of *Ignatzschineria* spp. bacteremia cases in persons experiencing homelessness, Vancouver, British Columbia, Canada, 2025. A) Bilateral leg wounds at time of admission; B) close-up of myiasis in exposed right tibialis anterior tendon.

Case 1 involved a 49-year-old man, a PEH with active substance use disorder (SUD) and chronic bilateral leg wounds, who sought care for worsening pain, swelling, and wound myiasis (Figure 1, panel A, B). We collected 2 aerobic and 2 anaerobic blood cultures, and within 24 hours, all 4 collection bottles grew gram-negative bacilli. After an additional 24 hours, small gray colonies appeared on sheep blood agar and clear colonies appeared on MacConkey agar (Appendix Figure 2). Matrix-assisted laser desorption/ionization time-of-flight mass spectrometry (MALDI Biotyper; Bruker, https://www.bruker.com) identified the isolates as *I. larvae*, *I. indica*, and *Fusobacterium varium*. Whole-genome sequencing (WGS) confirmed the species as *I. larvae* and *I. indica* (Appendix Figure 3).

We conducted antimicrobial susceptibility testing by using gradient strip diffusion method for several antibiotics (Table). We referenced the 2025 Clinical and Laboratory Standards Institute breakpoints for other non-Enterobacterales for interpretation of MICs (*8*). The genotypic antimicrobial resistance prediction for the *I. larvae* isolate was concordant with phenotype for all tested antimicrobials (Table). The *I. indica* isolate did not grow for phenotypic testing, but no genotypic resistance was detected for antibiotics of interest.

**Table:** Antibiotic MICs as determined by gradient strip diffusion method for *Ignatzschineria larvae* reported for case-patient 1 (blood culture) and case-patient 2 (blood and wound culture), Vancouver, British Columbia, Canada, 2025

Antibiotic	MIC, μg/mL			Interpretation
	Case-patient 1 blood culture	Case-patient 2 blood culture	Case-patient 2 wound culture	
Amoxicillin/clavulanate	0.032	0.047	0.047	Susceptible
Ceftriaxone	<0.002	<0.002	<0.002	Susceptible
Ciprofloxacin	0.094	0.047	0.047	Susceptible
Meropenem	0.023	0.012	0.012	Susceptible
Piperacillin/tazobactam	<0.016	<0.016	<0.016	Susceptible
Trimethoprim/sulfamethoxazole	0.023	0.06	0.008	Susceptible

The patient underwent surgical debridement of both leg wounds and a split thickness skin grafting from the upper thigh. Computed tomography imaging of the right lower leg demonstrated periosteal reaction and irregularity of the underlying bone cortex. He was treated for osteomyelitis with 6 weeks of amoxicillin/clavulanate. After antimicrobial therapy, the leg wounds healed well and showed healthy granulation tissue. We performed no repeat imaging.

**Figure 2:**
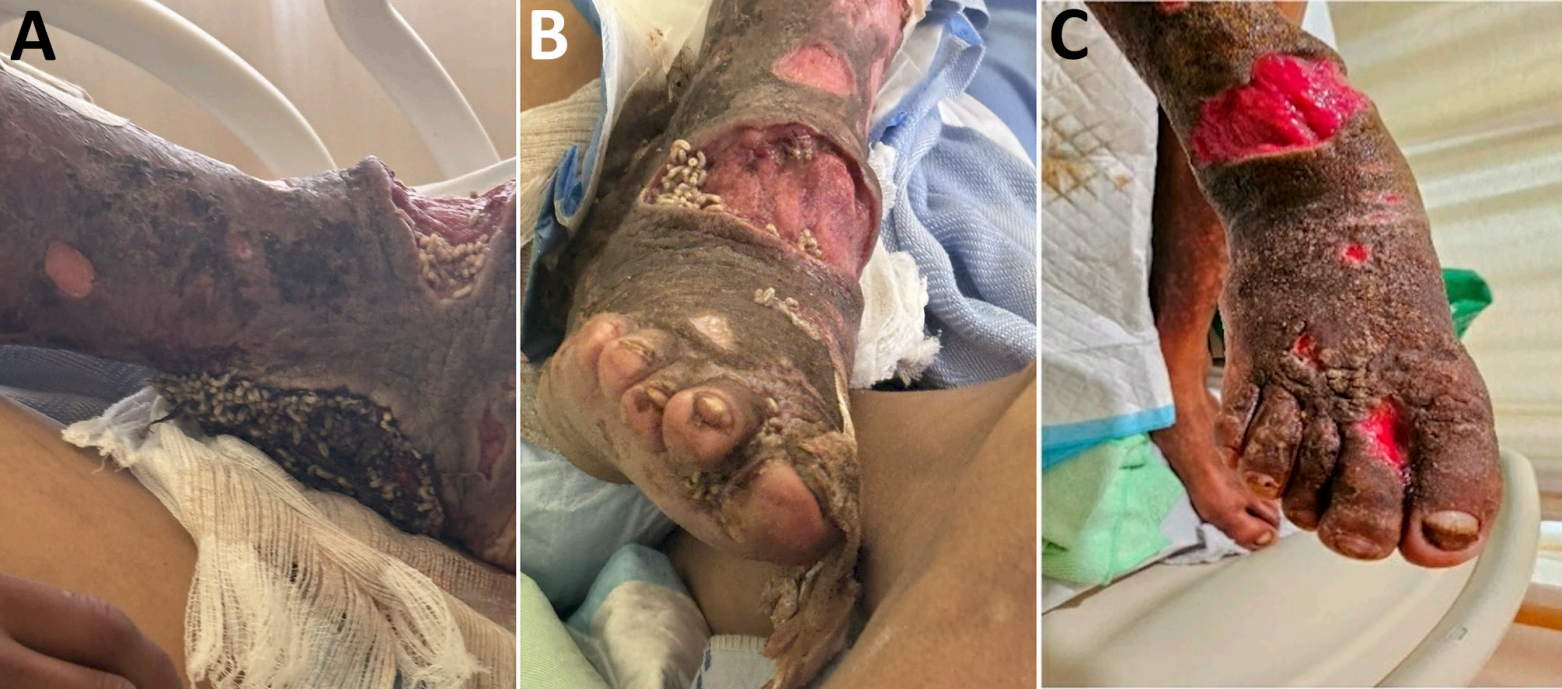
Leg wounds in case-patient 2 in pair of *Ignatzschineria* spp. bacteremia cases in persons experiencing homelessness, Vancouver, British Columbia, Canada, 2025. A, B) Lateral (A) and anterior (B) right leg wounds with visible myiasis at time of admission. C) Same wounds after 3 weeks of antibiotics and regular wound care.

Shortly after case-patient 1 was admitted to hospital, a second patient (case-patient 2), a 36-year-old man who was a PEH and SUD, sought care for fevers and an erythematous right shin containing necrotic wounds heavily infested with fly larvae (Figure 2, panel A, B). His blood cultures grew *I. larvae* and *Pasteurella multocida*, which we confirmed by using WGS. The genotypic antimicrobial resistance prediction was concordant with phenotype for all antibiotics tested (Table). His orthopedic and plastic surgery physicians recommended wound care without surgical intervention. The plan was to complete 6 weeks of ceftriaxone for osteomyelitis, but the patient self-initiated discharge before completing therapy.

**Figure 3:**
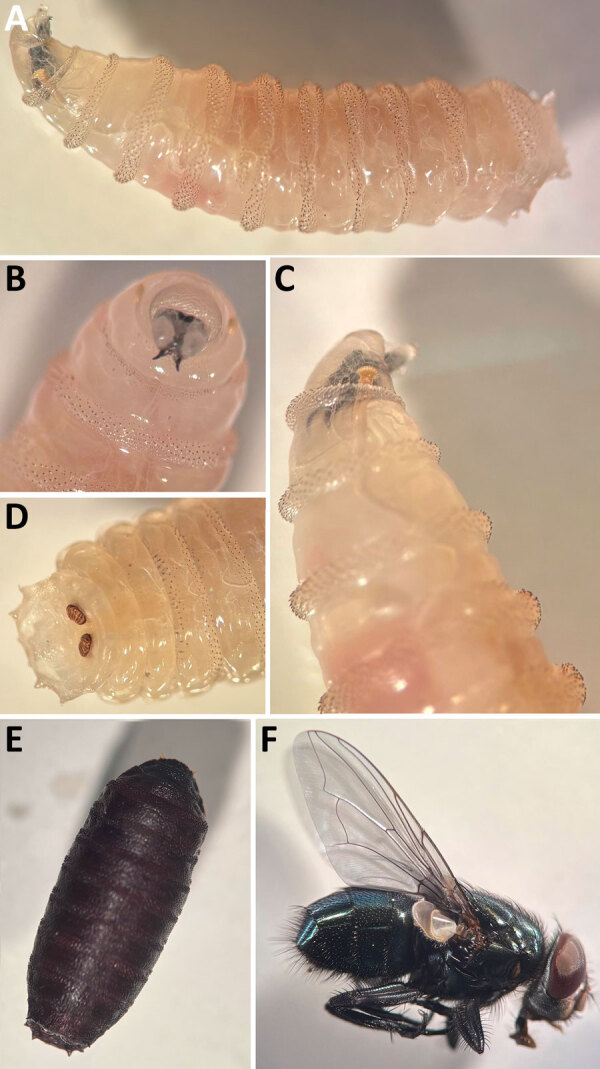
Development of black blow fly (*Phormia regina*) from larvae collected from case-patient 2 in pair of *Ignatzschineria* spp. bacteremia cases in persons experiencing homelessness, Vancouver, British Columbia, Canada, 2025. A–D) Third instar. E) Pupa. F) Adult. Key features of *P. regina* larvae include segmental spines (A), cephalopharyngeal skeleton (B), anterior spiracle (C), and peritremes (D).

When case-patient 2 sought care, we recognized a pattern of myiasis-associated bacteremia. We collected fly larvae from the patient in a sterile container, stored them on blood agar, and observed them over time to document their development into adult flies (Figure 3). The third instar larvae (9–12 mm) were creamy white with distinct bands along the body covered in short spines. The prothoracic spiracles had >10 openings. We observed 2 incomplete peritremes on the posterior with three inner slits directed toward the median line ventrally. The adult flies (10–12 mm) were metallic green. Considered together, those features were suggestive of the Calliphoridae family and the *Phormia* genus (*9*). We confirmed the fly to be *P. regina* by sequencing the cytochrome c oxidase subunit I gene using universal primers (Appendix Figure 4) (*10*). To confirm the presence of *Ignatzschineria* spp. in the fly larvae, we washed a larva 3 times in phosphate-buffered saline, macerated the anterior portion of the larva in phosphate-buffered saline, and subjected the homogenate to mechanical disruption with glass beads before DNA extraction and 16S rRNA sequencing. We uploaded sequencing data to the CZ ID platform (https://czid.org) for metagenomic analysis (S.E. Simmonds et al., unpub. data, https://doi.org/10.1101/2024.02.29.579666). We identified *Ignatzschineria* spp. at a 16S rRNA read abundance of 0.1% from the larva bacterial microbiome, providing molecular evidence of vector colonization.

## Conclusions

We have established a relationship between *P. regina* fly wound myiasis and *I. larvae* bacteremia by identifying *P. regina* flies as the probable causative agent of myiasis, isolating *Ignatzschineria* bacteria in patients’ blood cultures, and detecting the organism within the larval microbiome. Our findings fulfill the criteria for sequence-based determination of causation as proposed by Fredricks and Relman (*11*). Moreover, the larval microbiome results were consistent with the findings of Deguenon et al. (*12*), who identified *Ignatzschineria* bacteria in the microbiome of wild *P. regina* flies. Because *P. regina* blow flies are ubiquitous and widely distributed across North America, our results suggest that exposure risk could be far broader than previously assumed.

Both patients were at heightened risk for *Ignatzschineria* bacteremia because of their PEH status, SUD, chronic wounds, and competing health and social priorities (i.e., resuming everyday life versus receiving treatment and follow-up) (*13*). We hypothesize that the complexity of wounds and the magnitude of infestation might increase the risk for *Ignatzschineria* infection. Those cases also illustrate the value of a One Health perspective, in which human vulnerability, environmental exposure, and interactions with urban flies converge to create conditions for pathogen transmission. Clinicians should maintain a high index of suspicion and obtain blood cultures in patients with wound myiasis and systemic symptoms.

Accurate identification of *Ignatzschineria* bacteria remains a diagnostic challenge in the clinical microbiology laboratory. In several cases, *Ignatzschineria* isolates were initially misidentified as other bacteria using phenotypic and molecular methods (*7*,*13*). We observed that the Bruker MALDI Biotyper successfully identified *I. indica* and *I. larvae/ureiclastica* but could not differentiate between *I. larvae* and *I. ureiclastica* because of the high degree of genetic similarity. Of note, the VITEK MS version 3.2 knowledge base (bioMérieux, https://www.biomerieux.com) does not include *Ignatzschineria* species. Most case reports have relied on molecular methods such as 16S rRNA gene sequencing for genus identification, but only WGS can truly provide species-level resolution (*14*). Those findings highlight a broader diagnostic gap in detecting emerging zoonotic pathogens and demonstrate the value of WGS for achieving species-level resolution.

Information on the antimicrobial susceptibility of *Ignatzschineria* is currently limited. Reported testing methods include disk diffusion, automated instruments, gradient strip diffusion, or some combination of those methods (*1*). Most published cases demonstrate that *Ignatzschineria* bacteria are susceptible to β-lactam antibiotics, which comprise the mainstay of therapy (*1*). Treatment typically includes appropriate antimicrobial therapy combined with larvae removal, wound care, debridement, amputation, or some combination of these interventions (*1*,*7*). Overall, patient outcomes have been favorable when both infection and underlying wounds are addressed (*13*).

Appendix Additional information about *Phormia regina* fly as vector for *Ignatzschineria* spp. bacteremia in persons experiencing homelessness, Canada, 2025.
