# Supplementary material for: Phormia regina Fly as Vector for Ignatzschineria spp. Bacteremia in Persons Experiencing Homelessness, Canada, 2025
Source: Emerg Infect Dis. 2026 Jul;32(7):1150–4. doi: 10.3201/eid3207.251914 (PMC13322419; doi:10.3201/eid3207.251914)
Supplement: Appendix — Additional information about Phormia regina fly as vector for Ignatzschineria spp. bacteremia in persons experiencing homelessness, Canada, 2025. [file 25-1914-Techapp-s1.pdf]

Article DOI: https://doi.org/10.3201/eid3207.251914

EID cannot ensure accessibility for supplementary materials supplied by authors. Readers who have difficulty accessing supplementary content should contact the authors for assistance.

Phormia regina as Vector for *Ignatzschineria spp.* Bacteremia in Persons Experiencing Homelessness, Canada, 2025

Appendix

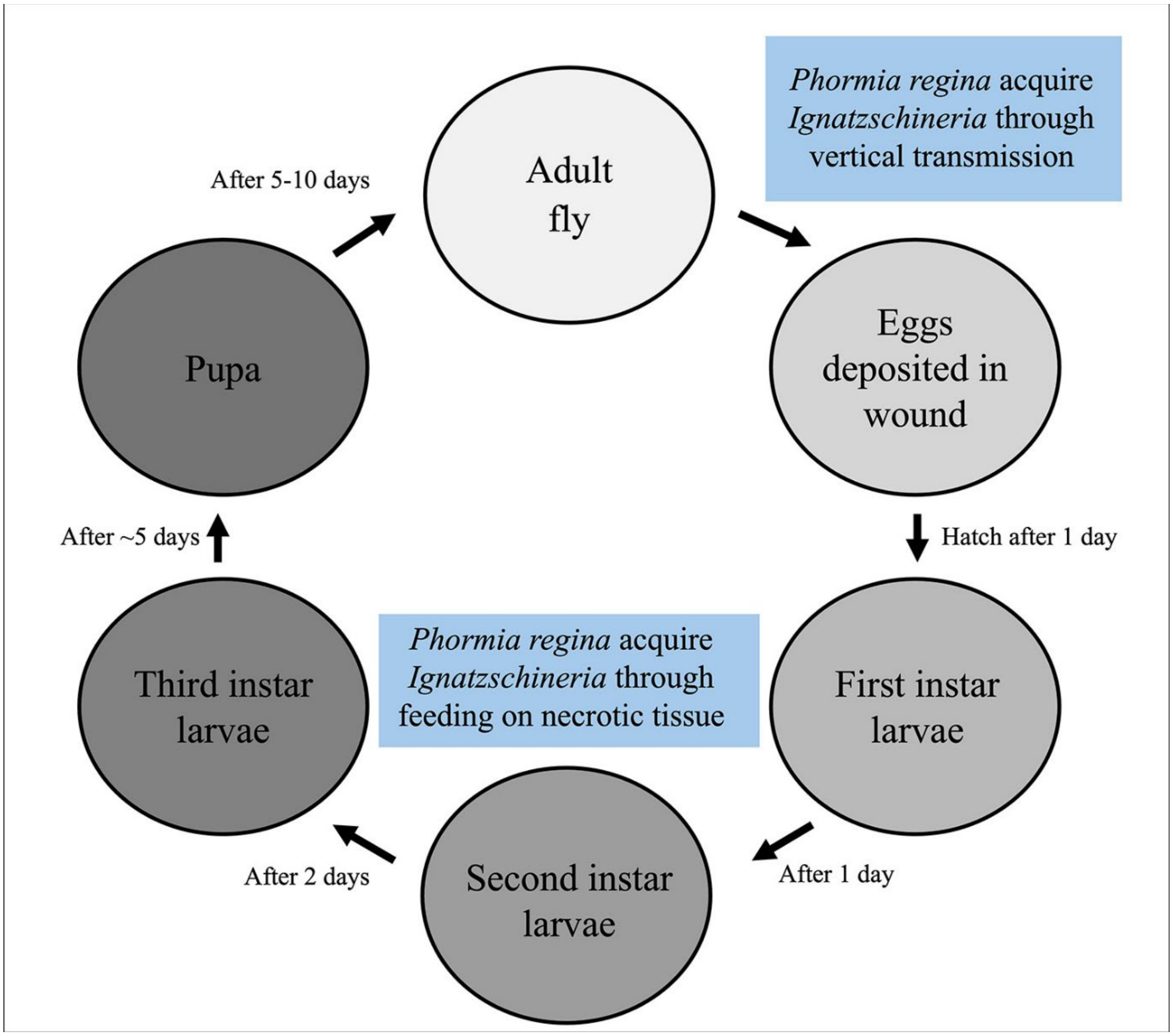

Appendix Figure 1. Life cycle of *Phormia regina* and proposed transmission points of *Ignatzschineria* larvae in wound myiasis. There are two main ways for *P. regina* to acquire *Ignatzschineria*: through vertical transmission and through feeding on necrotic and decaying tissue.

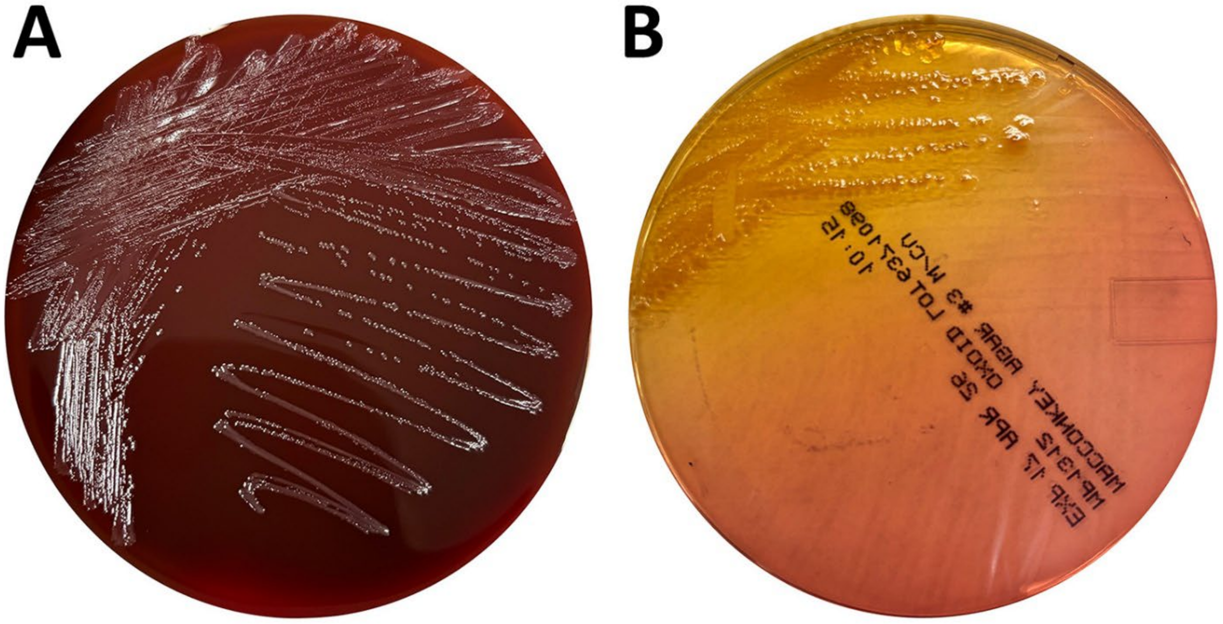

Appendix Figure 2. Growth of pure *Ignatzschineria larvae* on sheep blood agar (A) and MacConkey agar (B) after 24 hours at 37°C under aerobic conditions.

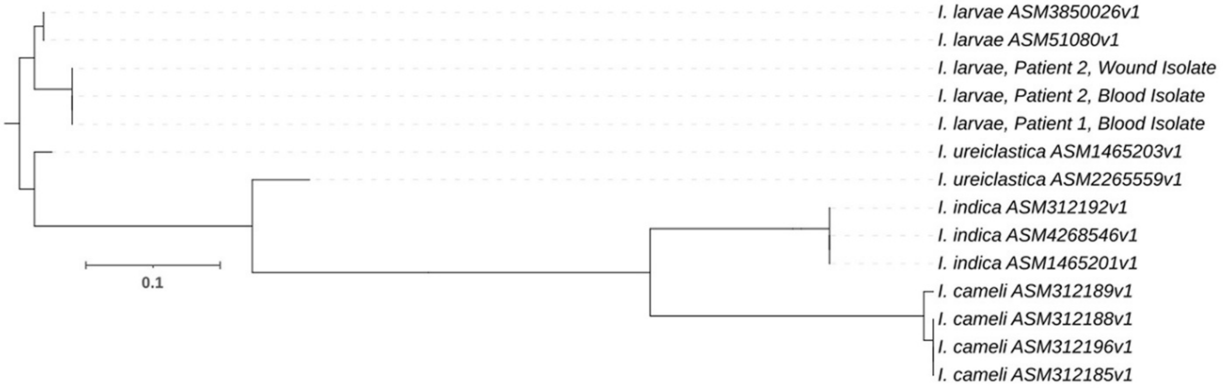

Appendix Figure 3. Whole-genome core phylogenetic analysis of *Ignatzshineria* clinical isolates. This core-genome phylogenetic tree, derived from multi-sequence alignment of single-copy orthologs, illustrates the evolutionary relationships of the *I. larvae* isolates (from Patient 1 and Patient 2) in the context of publicly available *Ignatzshineria* reference genomes. The tree was constructed using Buscogeny (Webster & Chapman, 2024) based on core single-copy orthologs identified by BUSCO. Tree scale bar represents 0.1 substitutions per site.

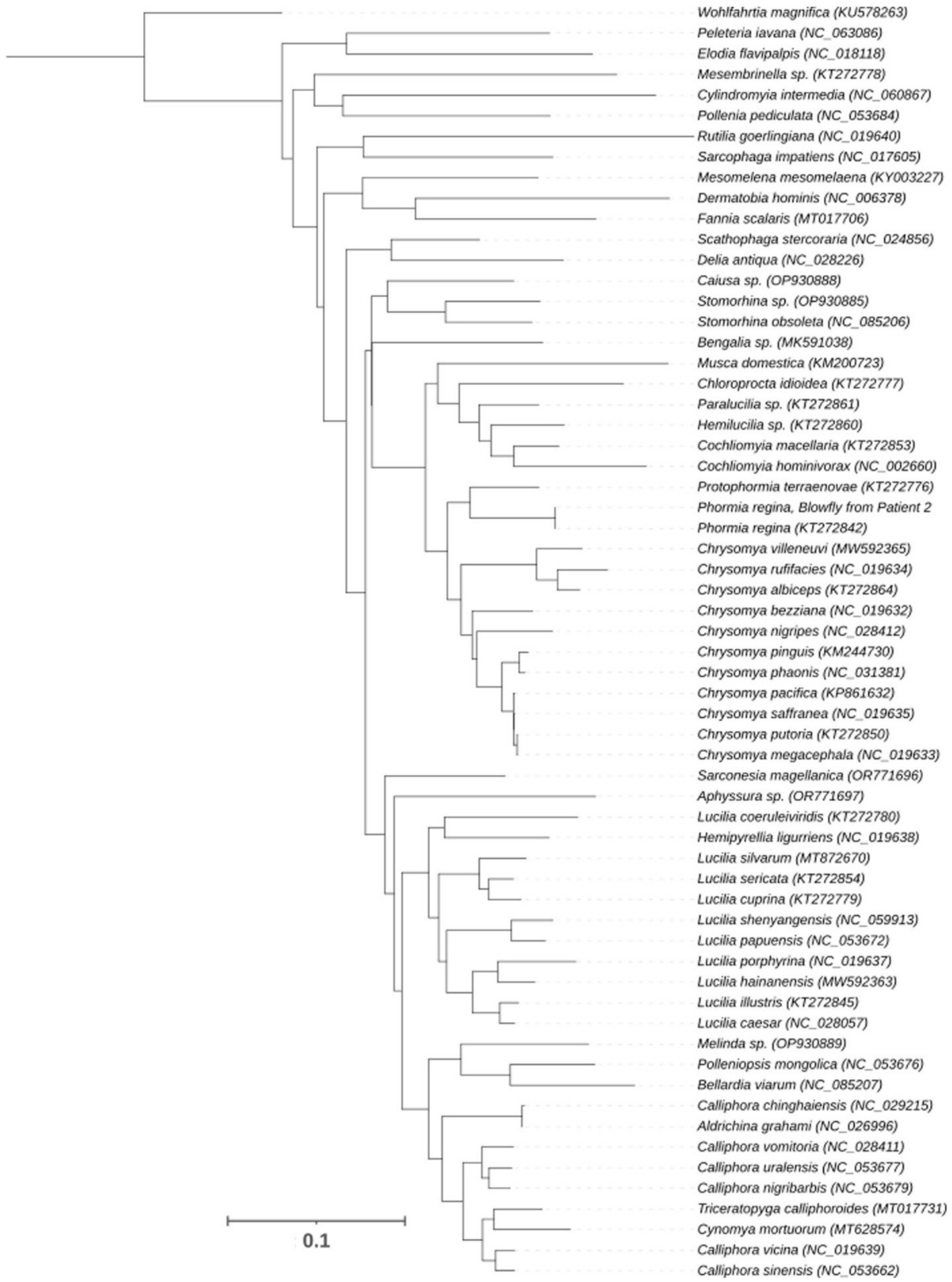

Appendix Figure 4. Phylogenetic analysis of COX1 amino acid sequence. This maximum-likelihood phylogenetic tree illustrates the relationship of the blow fly isolate recovered from case-patient 2 among 63 representative species within the family Calliphoridae and related calyptrate flies. Identification was based on the cytochrome c oxidase subunit I (*COX-1*) gene. Sequences were aligned using MAFFT version 7 (with --auto parameter), and the tree was reconstructed using IQ-TREE 2. The tree scale bar indicates 0.1 substitutions per site.
